# Acute Generalized Exanthematous Pustulosis Induced by Etanercept: Another Dermatologic Adverse Effect

**DOI:** 10.1155/2013/601412

**Published:** 2013-03-20

**Authors:** Mukaddes Kavala, Ilkin Zindancı, Zafer Türkoglu, Burçe Can, Emek Kocatürk, Serkan Senol, Filiz Topaloglu

**Affiliations:** ^1^Department of Dermatology, Istanbul Medeniyet University, SB Istanbul Goztepe Training and Research Hospital, 34732 Istanbul, Turkey; ^2^Department of Pathology, Istanbul Medeniyet University, SB Istanbul Goztepe Training and Research Hospital, 34732 Istanbul, Turkey

## Abstract

Acute generalized exanthematous pustulosis (AGEP) is a skin eruption that is primarily drug induced and characterized by the formation of numerous acute and sterile pustules on an erythematous background as mentioned by Weinblatt et al. (1999). We present a case of AGEP, following administration of etanercept, an antitumour necrosis factor alpha (TNF-**α**) antibody, in a patient with psoriasis. Recognition of this reaction pattern is important given the frequent reliance on etanercept in treating psoriasis.

## 1. Case

**Figure 1:**
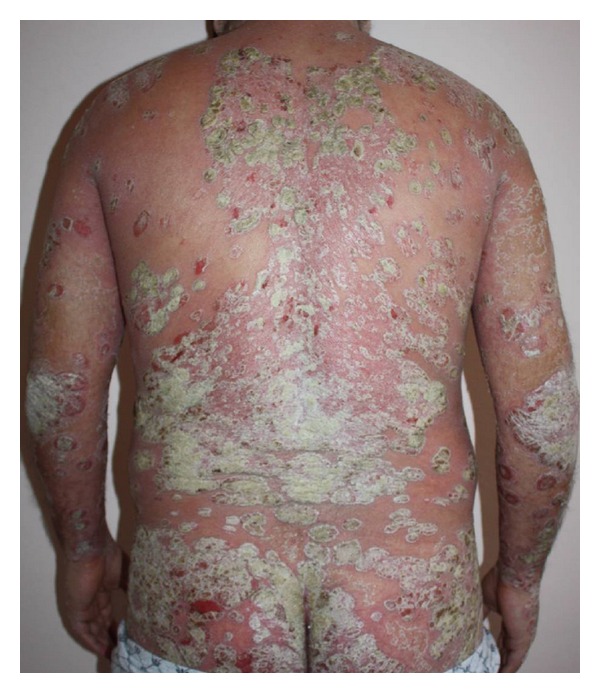
Severe plaque type psoriasis involving the face, scalp, trunk, and limps.

**Figure 2:**
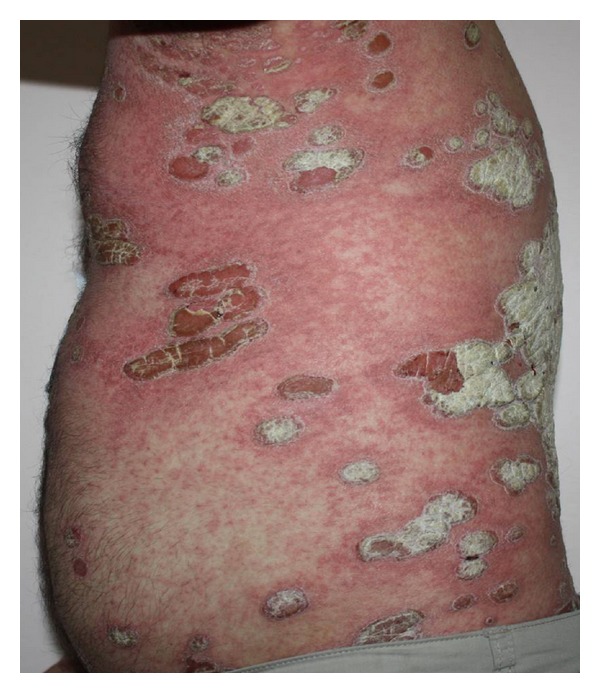
Generalized erythoderma characterized by tiny pustules which has developed 4 days after the second injection.

**Figure 3:**
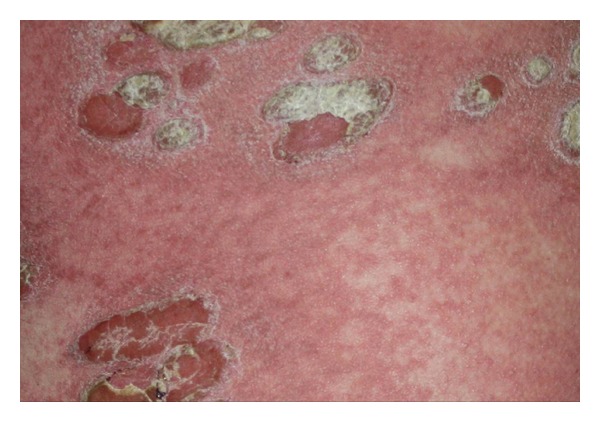
Closer view to psoriasis plaques, erythematous rash, and tiny pustuloses.

**Figure 4:**
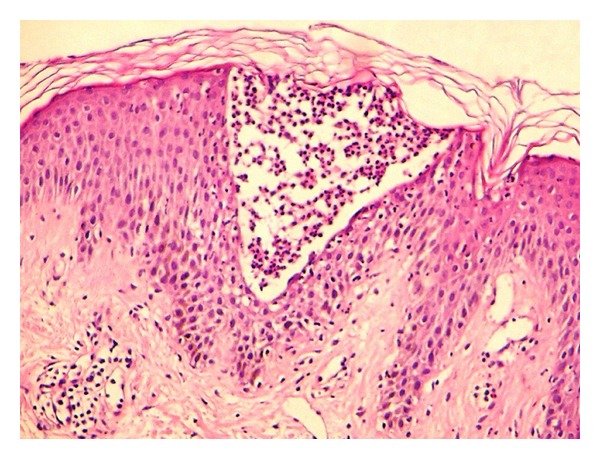
Subcorneal and intraspinous collections of neutrophils with mild epidermal oedema (H and E × 40).

A 29-year-old, healthy man was admitted to our department with a 6-year history of psoriasis. His psoriasis had been treated with narrowband ultraviolet B phototherapy, psoralen plus ultraviolet A photochemotherapy, acitretin, and topical steroids. He also was previously treated with cyclosporin for 4 months. He discontinued cyclosporin secondary to adverse effects, and subsequently started oral methotrexate at a dose of 20 mgr weekly with minimal improvement of his psoriasis. At the time of admission, he presented with severe plaque type psoriasis involving the face, scalp, trunk, and limps (Figure 1). We decided to start etanercept 50 mgr subcutaneously twice a week. One day after initiation of etanercept therapy, he developed pruritic, erythematous halo around the psoriasis lesions that evolved into widespread maculopapular erythematous rash on the second day. It was decided to continue etanercept and treatment with oral antihistaminics and topical corticosteroids. Following the second injection after 4 days, the rash progressed with the development of generalized erythoderma characterized by tiny pustules (Figures 2 and 3). His general condition was good, and there was no systemic symptoms that may accompany skin eruptions including fever, leukocytosis, reduction in creatinine, or elevation of aminotransferases. Bacterial and fungal cultures of the patient's blood and pustules were negative. Acute generalized skin rash was attributed to etanercept, and, subsequently, a skin biopsy was taken from the pustular lesions. Histopathologic examination revealed subcorneal and intraspinous collections of neutrophils with mild epidermal oedema (spongiosis) consistent with AGEP (Figure 4). Etanercept was discontinued, and the rash improved with systemic antihistaminics and topical corticosteroids treatment.

## 2. Discussion

Etanercept is a recombinant tumour necrosis factor alpha soluble receptor fused to the Fc fragment of IgG and is a potent inhibitor of inflammation [1]. Since etanercept is currently used for treating patients with plaque psoriasis and psoriatic arthritis, the incidence of inflammatory skin reactions has increased significantly. Etanercept is associated with a variety of dermatologic side effects. Injection site reactions are the most frequent cutaneous side effects for etanercept [2]. This has been reported in 37% of rheumatoid arthritis patients who received etanercept [3]. These patients may also present with erythema, pruritus, pain, and oedema and do not necessitate discontinuation of therapy [1]. A wide range of different skin lesions have been reported in controlled trials of etanercept such as urticaria [2], leukocytoclastic vasculitis [3], lichenoid reaction [4], erythema multiforme [5], Steven-Johnson syndrome, and toxic epidermal necrolysis. 

 A comprehensive overview of dermatologic adverse events of etanercept treatment for any indication described in the literature revealed that patients had approximately 65 different specific dermatologic adverse events of etanercept [6]. Of the 153 patients described in case reports, 38 presented with guttate, pustular, or plaque type psoriasis as dermatologic adverse events of etanercept. Thirty-one patients presented with skin infections of bacterial, viral, fungal, and parasitic origin. Malignant neoplasms in 15 patients, several cutaneous forms or symptoms of lupus in 19 patients, cutaneous vasculitis in 15 patients, and miscellaneous dermatologic adverse events in 35 patients have also been described with the use of etanercept. Nonspecified rash and urticaria were the most common miscellaneous dermatologic adverse events whereas alopecia, lichen planopilaris, lentigines, dermatomyositis, and granuloma annulare were reported only once or twice [6]. There are few reports of generalized skin eruption during the course of TNF-*α* inhibitors. Beuthien et al. [7] reported a case of generalized rash due to adalimumab in a female patient who developed an erythema multiforme-like skin rash after the sixth adalimumab injection. Dalmau et al. [8] reported two cases of adalimumab induced generalized erythematous rash, confirmed by the biopsies, one week after the first injections.

Kucharekova et al. [9] reported the first case of generalized pustulosis as an adverse effect of adalimumab. One patient who developed severe urticaria and angiedema, followed by hypotension, after the seventh injection has also been described with the use of adalimumab [10]. Only one case of generalized symptomatic maculopapular rash due to etanercept has been reported to date [11]. This occurred in a 70-year-old female patient with rheumatoid arthritis who developed a widespread inflamed, itchy, erythematous macular lesions after the fourth etanercept injection. Her biopsy was consistent with a drug-induced dermatitis, and the rash improved within 2 weeks after discontinuation of therapy. In contrast with the previous case, our patient developed AGEP triggered by etanercept. Although the exact pathomechanisms underlying AGEP have not been elucidated, it appears to be a T-cell process [12]. Both drug-specific CD4 and CD8 reactions occur, leading to the production of IL-8 and IL-5. It has been suggested that AGEP represents a type of delayed hypersensitivity reaction with predominantly neutrophils being activated [13]. Pustular dermatitis has been reported previously as a possible adverse effect during anti-TNF-*α* therapy. In this report, it was suggested that TNF-*α* inhibitor therapy may produce aberrant INF-*α* expression at the tissue level in predisposed individuals and thus promote psoriasis lesion induction similar to infection or injury [14]. 

Generalized pustular psoriasis should be considered in differential diagnosis of AGEP. These two entities can imitate each other clinically, and there is no specific finding in histopathologic evaluation. Eosinophils in the dermis or pustules, necrotic keratinocytes, neutrophilic dermal infiltration, absence of epidermal psoriasiform changes, and dilated blood vessels can be considered as findings of AGEP as in our case [15].

In our case, the immunologic mechanisms behind AGEP as a complication of etanercept is not known. It is possible that the effect of TNF blockade, combined with other precipitating factors result in dysregulation of T cells in the epidermis with the development of AGEP. 

The case presented here indicates another example of a potential adverse effect of etanercept. We assume that as the use of TNF-*α* inhibitor therapy increases, the clinicians will recognize more cutaneous adverse reactions than previously suspected.
